# Personal

**Published:** 1904-09

**Authors:** 


### PERSONAL.

Dr. John H. Grant, of Buffalo, was elected surgeon of the United Spanish War Veterans at the state encampment recently held at Albany. We publish elsewhere in this edition an important paper from Dr. Grant's pen, read at the semiannual meeting of the Medical Society of the County of Erie in June, entitled, "Adulteration of Food and Food Products." This is a subject with which every physician should familiarise himself and Dr. Grant handles it from the standpoint of a master.

---

Dr. Augustus G. Pohlman, of Ithaca, a graduate of the University of Buffalo in 1900, has resigned an instructorship at Johns Hopkins University to accept an appointment as assistant professor of anatomy in the University of Indiana, located at Bloomington in that state.

---

Dr. Frederick H. Millener, of Buffalo, has established a laboratory in connection with his nose and throat work, for the diagnosis and treatment of diseases and injuries by means of the various forms of light, such as Finsen's, *x*-ray and ultra violet light. Office, 724 Main street.

---

Dr. Sidney D. Wilgus, a graduate of the University of Buffalo in 1895, has been appointed by the state lunacy commission as chief examiner of the state board of alienists. This board, in coöperation with the United States examiners, will examine all emigrants who arrive at the port of New York, who are suspected of epilepsy, imbecility, or any form of insanity. All persons afflicted in this manner are deemed unfit to remain and are returned to their own country. Dr. Wilgus will receive $5,000 a year and has as assistants, Dr. George D. Campbell, of New York, and Dr. W. E. Sylvester, of College Point.

---

Dr. Edward Clark, of Buffalo, assistant commissioner of health, who has been for some time under treatment at Dr. Dunham's private hospital, suffering from multiple neuritis, is reported convalescent. Dr. Clark expects to resume his practice in a short time.

---

Dr. Eugene Smith, of Detroit, has returned from his annual European visit and resumed his ophthalmological practice. He read a paper before the American Academy of Ophthalmology and Oto-laryngology at its annual meeting held at Denver, August 24-26, 1904, entitled, "Removal of the Anterior Capsule and the Hypodermatic use of Morphia in simple Extraction."

---

Dr. Bernard Panzer, of Vienna, was recently the guest of Dr. George F. Cott, in this city. Dr. Panzer addressed the Roswell Park Medical Club on "Diseases of the Accessory Sinuses of the Nose in Children."

